# Urban chromoblastomycosis: a diagnosis that should not be neglected^☆^

**DOI:** 10.1016/j.abd.2022.01.013

**Published:** 2023-02-04

**Authors:** Jessica Lana Conceição e Silva Baka, Gabriela Giraldelli, Andrea Reis Bernardes-Engemann, Carlos Baptista Barcaui, Rosane Orofino-Costa

**Affiliations:** a Service of Dermatology, Hospital Universitário Pedro Ernesto, Universidade do Estado do Rio de Janeiro, Rio de Janeiro, RJ, Brazil; b Laboratory of Mycology, Hospital Universitário Pedro Ernesto, Universidade do Estado do Rio de Janeiro, Rio de Janeiro, RJ, Brazil

Dear Editor,

Chromomycosis, a neglected disease with a strong rural profile, is caused by melanized geophilic fungi. Infection occurs after traumatic inoculation into the skin, and *Fonsecaea pedrosoi* is the most common agent.^1^, ^2^, ^3^

This case report describes two cases of chromoblastomycosis in inhabitants of an urban area, and their epidemiological and therapeutic data.

## Case reports

**Figure 1:**
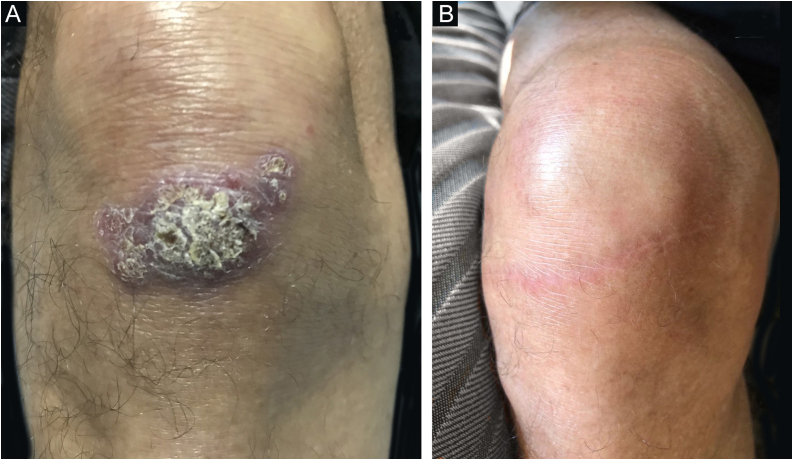
Case 1. (A) Asymptomatic verrucous plaque, measuring approximately 5.0 × 3.0 cm, with well-defined borders, and small areas intermingled with blackened dots and satellite lesions, located on the extensor surface of the right knee. (B) Three years after surgical excision with safety margins, both superficial and deep.

Case 1 A 69-year-old healthy male physician who lives in the southern zone of the municipality of Rio de Janeiro reported that after falling from his bicycle during a tropical storm in Parque Lage, an urban forest inside the city of Rio de Janeiro, an open wound appeared on his right knee, caused by trauma from a tree branch. The lesion was a plaque with a verrucous appearance, well-defined borders and small satellite lesions (Fig. 1).

**Figure 2:**
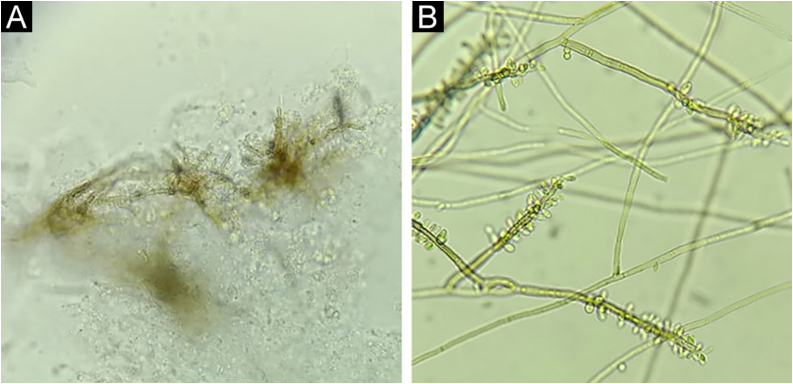
Case 1. (A) Septate, brownish hyphae (“Borelli spiders”). Direct microscopy, KOH 20% + DMSO, ×40. (B) Colony micromorphology showing brownish, septate hyphae, from which conidiophores with elongated conidia at their extremities arise, giving the appearance of a gnarled staff. *Rhinocladiella* spp., lactophenol, ×40.

Direct mycological examination (DME) showed brownish hyphae (Fig. 2A) in addition to muriform bodies, and *Rhinocladiella spp.* (Fig. 2B) was isolated in the culture.

He underwent surgical excision of the lesion with wide margins. Two weeks before the intervention, itraconazole 200 mg/day was started for six months, followed by 100 mg/day for four months. There was no recurrence or complications after three years of follow-up (Fig. 1).

**Figure 3:**
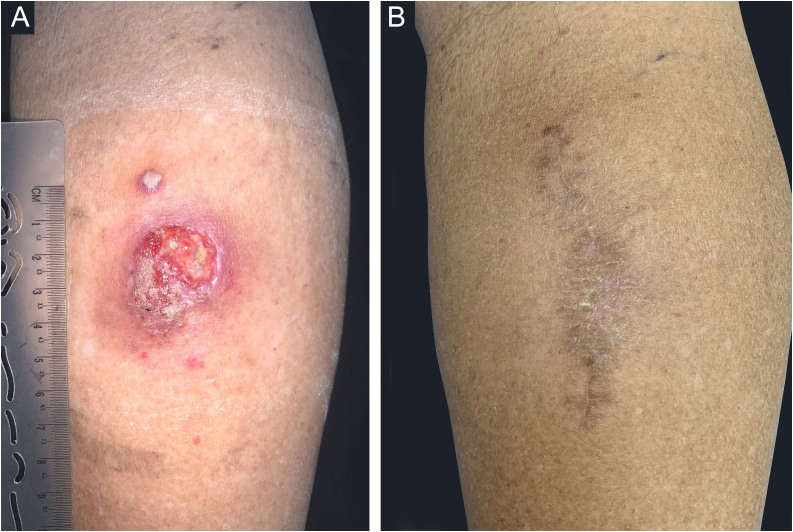
Case 2. (A) An infiltrated, well-delimited plaque, with ulceration and purulent background, measuring approximately 5.0 cm, on the posterior right calf and a nearby verrucous lesion, measuring approximately 0.5 cm; (B) Eight months after follow-up.

Case 2 A 67-year-old male patient who lived in the northern zone of the municipality of Rio de Janeiro worked as a manager. Currently, he presented with diabetes mellitus and systemic arterial hypertension, taking losartan, atenolol and metformin. A year ago, a papule appeared on his right calf, progressing into ulceration with purulent discharge (Fig. 3A). He was treated with topical and systemic antibiotics, and topical antifungals and corticosteroids, with no improvement. He denied previous trauma.

DME was positive for chromoblastomycosis, and *Fonsecaea spp.* was isolated in the culture. Itraconazole 200 mg/day was started. Two months later, satellite lesions were observed, as well as lesion enlargement. Wide surgical excision was performed, with the maintenance of oral itraconazole for eight months. There was no recurrence after eight months of follow-up (Fig. 3B).

## Discussion and conclusion

Although 90% of cases of chromoblastomycosis are described in individuals with rural activities,^1^, ^4^, ^5^ the two patients described herein did not report this type of activity.

Around the world, there are still cities with preserved native nature, such as the Floresta da Tijuca, the largest urban forest in the world, located in the municipality of Rio de Janeiro. The search for healthy outdoor activities is becoming increasingly frequent in urban centers and diseases related to nature agents must be taken into account in the differential diagnosis, including chromoblastomycosis mainly the verrucous lesions. The location and size of the lesions suggested the moderate verrucous type of the disease in both cases.^1^

The isolation of *Rhinocladiella spp.* is another unusual finding since there are few published reports of this species in Brazil.^2^, ^5^, ^6^ The presence of hyphae in DME may indicate greater potential for invasion with the production of pro-inflammatory cytokines (TNF-α, IL1-β, IL-6).^7^, ^8^

The best treatment approach is to combine an oral antifungal initially, most often itraconazole, with extensive surgical excision.^4^, ^9^, ^10^ Cryosurgery is another recommended adjunctive treatment.^1^, ^4^, ^10^ These two patients were cured with no recurrence and no impairment of their social and professional lives.

The authors intend to emphasize the importance of the differential diagnosis in patients with urban activity in case of diseases linked to rural activity, preventing unnecessary treatments and chronicity. Attention to the clinical and laboratory diagnosis and the early treatment can increase the chances of cure.

## Financial support

None declared.

## Authors' contributions

Jessica Lana Conceição and Silva Baka: Design and planning of the study; drafting of the original version; editing and critical review of the manuscript; participation in the therapeutic conduct; critical review of the literature and manuscript; approval of the final version of the manuscript.

Gabriela Giraldelli: Design and planning of the study; drafting of the original version; editing and critical review of the manuscript; participation in the therapeutic conduct; critical review of the literature and manuscript; approval of the final version of the manuscript.

Andrea Reis Bernardes-Engemann: Design and planning of the study; drafting of the original version; editing and critical review of the manuscript; participation in the therapeutic conduct; critical review of the literature and manuscript; approval of the final version of the manuscript; effective participation in research orientation.

Carlos Baptista Barcaui: Design and planning of the study; drafting of the original version; editing and critical review of the manuscript; participation in the therapeutic conduct; critical review of the literature and manuscript; approval of the final version of the manuscript; effective participation in research orientation.

Rosane Orofino-Costa: Design and planning of the study; drafting of the original version; editing and critical review of the manuscript; participation in the therapeutic conduct; critical review of the literature and manuscript; approval of the final version of the manuscript; effective participation in research orientation.

## Conflicts of interest

None declared.
